# Phenomenological experience personality profile: A test to identify the affective dimensions of psychopathology in the context of precision psychotherapy

**DOI:** 10.1192/j.eurpsy.2021.1217

**Published:** 2021-08-13

**Authors:** R. Sperandeo, V. Cioffi, L. Mosca, B. Muzii, N. Maldonato

**Affiliations:** 1 Phenomena Research Group, Sipgi, Post graduate School of Psychotherapy, Torre Annunziata, Naples, Italy; 2 Department Of Humanistic Studies, University of Naples Federico II, Naples, Italy, Naples, Italy; 3 Neurosciences And Reproductive And Odontostomatological Sciences, University of Naples Federico II, Naples, Italy, Naples, Italy

**Keywords:** Personality, psychopathology, test, Artificial Intelligence

## Abstract

**Introduction:**

Artificial intelligence algorithms are increasingly used to highlight refined qualifiers of pathologies and to build treatment protocols based on them. These possibilities open up new perspectives for personalized interventions in psychotherapy. The affective neurosciences that link psychopathological phenomena to the hypersensitization of emotional systems are an excellent field of application of deep learning algorithms

**Objectives:**

In this contribution we present the standardization of a psychodiagnostic test that can be analyzed with a deep learning algorithm for the development of personalized treatments for depressive disorders in a perspective of precision psychotherapy

**Methods:**

Previously we have constructed a psychodiagnostic test that correlates the psychopathological characteristics to the emotional systems described in affective neuroscience. The construction of this test was carried out with the use of a neural network that discriminated 161 items from a pull of 300 psychopathological and character descriptions. In the present work, the 161 selected items were compared, in a sample of 600 subjects, with the measurement of sadness described in the Panksepp model. Comparation was performed with linear and non-linear statistical analysis methods.

**Results:**

The items emerging from the statistical analyzes as strongly indicative of a hypersensitivity of the sadness system outline a psychopathological profile for which it is possible to adapt specific psychotherapeutic treatment protocols.

**Conclusions:**

In future prospect, neurobiological and psychophysiological variables such as heart rate variability, skin conductance and activity of the areas of the cortex, measured with a scanner of the near infrared photons, will be correlated to these descriptors of psychopathology.

